# Halogen and Hydrogen Bonding Interplay in the Crystal Packing of Halometallocenes

**DOI:** 10.3390/molecules23112959

**Published:** 2018-11-13

**Authors:** Karina Shimizu, João Ferreira da Silva

**Affiliations:** Centro de Química Estrutural, Instituto Superior Técnico, U. Lisboa, Av. Rovisco Pais, 1049-001 Lisboa, Portugal; karina.shimizu@tecnico.ulisboa.pt

**Keywords:** supramolecular chemistry, noncovalent interactions, halogen bonding, database analysis, ab initio calculations

## Abstract

This paper focuses in the influence of halogen atoms in the design and structural control of the crystal packing of Group VIII halogenated metallocenes. The study is based on the present knowledge on new types of intermolecular contacts such as halogen (X⋯X, C-X⋯H, C-X⋯π), π⋯π, and C-H⋯π interactions. The presence of novel C-H⋯M interactions is also discussed. Crystal packings are analysed after database search on this family of compounds. Results are supported by ab initio calculations on electrostatic charge distributions; Hirshfeld analysis is also used to predict the types of contacts to be expected in the molecules. Special attention is given to the competition among hydrogen and halogen interactions, mainly its influence on the nature and geometric orientations of the different supramolecular motifs. Supramolecular arrangements of halogenated metallocenes and Group IV di-halogenated bent metallocenes are also compared and discussed. Analysis supports halogen bonds as the predominant interactions in defining the crystal packing of bromine and iodine 1,1′-halometallocenes.

## 1. Introduction

Several reports of what could be considered weak noncovalent interactions between molecules are found in the literature as early as the end of the 19th century [1]. In the beginning of the 20th century they were identified as van der Waals forces [2,3,4] and hydrogen bonds [5,6]; it was Pauling who used for the first time the term “hydrogen bond” in its 1931 paper on the nature of chemical bonds [7]. On his work “Nature of the Chemical Bond” [8] he states, “Under certain conditions an atom of hydrogen is attracted by rather strong forces to two atoms instead of only one, so it may be considered to be acting as a bond between them. This is called a hydrogen bond”. He also assumed an electrostatic character for hydrogen bonds D-H⋯A, that should result in their formation only when the donor atom (D) and the acceptor (A) were electronegative atoms like N, O, F, Cl, Br and I. 

The involvement of other donor atoms was first proposed by Pimentel and McClellan in 1960 [9], further reinforced in 1993 by Steiner and Saenger [10], that defined hydrogen bond as “any cohesive interaction X-H⋯A where H carries a positive charge and A a negative charge and the charge on X is more negative than in H”. This definition includes donor atoms like C, P, As, as well as π acceptors, creating the possibility for the existence of strong and weak hydrogen bonds, depending on the properties of the atoms involved [11]. 

More recently a report of the IUPAC Task Group on Categorizing Hydrogen Bonding and Other Intermolecular Interactions [12] presented a very comprehensive analysis of examples of hydrogen bonds involving various types of donor and acceptor atoms, together with a list of their characteristics and the criteria used as confirmation for their existence. They also briefly examined the various types of contributions for the formation of hydrogen bonds, ranging from electrostatic to dispersion and charge transfer interactions. Among the criteria listed for the identification of hydrogen bonds were spectroscopic and computational indications, but considerable relevance was given in the report to crystallographic evidences. The authors consider directionality (that strongly affects crystal packing) as one of the most relevant characteristics of a hydrogen bond, differentiating it from simple electrostatic or dispersion interactions. The commission concludes by proposing a definition for hydrogen bond [13], based on the one presented by Pimentel and McClellan [9], but stressing that the donor atom should be more electronegative than hydrogen: “The hydrogen bond is an attractive interaction between a hydrogen atom from a molecule or a molecular fragment X–H in which X is more electronegative than H, and an atom or a group of atoms in the same or a different molecule, in which there is evidence of bond formation”.

We decided to study the presence of hydrogen bonds and other types of noncovalent interactions in a family of compounds (halometallocenes), not very complex in structure to avoid complicated supramolecular frameworks, but containing a diverse set of donor-acceptor groups, such as halogen atoms, aromatic rings and, as single hydrogen donors, C-H bonds. It should be expected these groups to be involved not only in weak C-H⋯X hydrogen bonds and X⋯X halogen contacts (X = halogen atom), but also in C-X⋯π bonds and C-H⋯π interactions; the same aromatic rings can also participate in dispersion based π-π contacts. The presence of the metal atom can also enable these molecules to participate in a novel type of weak hydrogen bonds, C-H⋯M, found in similar compounds like orthorhombic biscyclopentadienyl ruthenium [14]. 

**Scheme 1:**
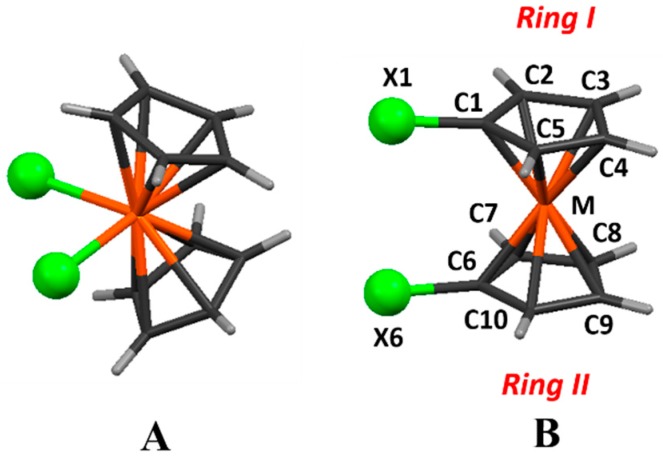
(**A**) di-halogenated bent metallocenes; (**B**) 1,1′-halometallocenes (the numbering is the one used in this text. Hydrogen atoms get their numbering from the respective carbon atoms. In the text and Tables ring C1–C5 will be mentioned as ring I, while ring C6–C10 will be ring II).

The halometallocenes are known for quite some time [15], but no systematic study has been elaborated about their crystal frameworks and the interactions in which they are based, mainly because by the time these compounds where first synthesized most of noncovalent interactions were not well known yet. It was decided to divide these compounds in two groups (Scheme 1): di-halogenated bent metallocenes (I) and 1,1′-halometallocenes (II), in an attempt to understand if the presence of the halogen atoms bonded directly to the metal or as substituents in the aromatic ring had different consequences on the supramolecular arrays formed by the molecules. The work in di-halogenated bent metallocenes has been published previously [16] and this paper will be focused in studying the 1,1′-halometallocenes and comparing the crystal frameworks of the two types of compounds.

Previous studies by various authors proved that the crystal packing of compounds containing halogen atoms is based in the competition between X⋯X and X⋯H contacts [16,17,18,19]. The relative importance of each type of interaction depends on two properties of halogen atoms: electronegativity and polarizability. While electronegativity decreases from fluorine to iodine, polarizability increases in the same order; therefore X⋯H bonds should be more relevant for fluorine and chlorine, whereas X⋯X contacts can be found more frequently with bromine and iodine.

Despite their high electronegativity halogens were considered poor acceptors in hydrogen bonds involving strong donors like N or O [17] due to their low polarizability and very contracted lone pairs (particularly in the case of fluorine) [20,21]. But in the absence of strong donors and acceptors, interactions like C-H⋯X-C were more frequent, even with fluorine [20,21,22].

**Scheme 2:**
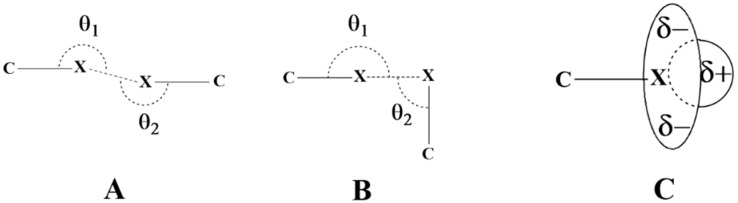
(**A**) Type I halogen interactions; (**B**) Type II halogen interactions; (**C**) Anisotropy of the electronic distribution around halogen atom.

Halogen contacts are relatively recent interactions [17,23,24,25,26,27]. The most recent classification (Scheme 2) divides them into three classes: type I, with 0° ≤ |θ1 − θ2 | ≤ 15°, type II, with 30° ≤ |θ1 − θ2|, while those with 15° ≤ |θ1 − θ2 | ≤ 30° were listed as quasi type I/type II interactions [28].

Even now the definition of “halogen bond” is a matter of debate. Type I and Type III contacts are essentially close packing Van der Waals interactions [28,29]. Only interactions like type II halogen contacts involving electron acceptor halogens atoms can be considered “halogen bonds” [18,30,31]. They comprise an interaction between an electropositive hole on the top of the halogen atom opposite to the C-X σ bond (a “σ-hole”) and a nucleophilic ring of another halogen, both results from the anisotropy of the electrostatic potential of the atom (Scheme 2C) [18,28,29,32,33]. The size of this hole depends on the polarizability of the atom, increasing from fluorine to iodine [20,34,35].

The model of “σ-hole” was first presented by Clark [33] applied to halogen bonding, as the result of the involvement in a covalent bond of a half-filled *p* orbital of an halogen, that created an electron deficiency in the outer lobe of the same orbital, associated to a positive electrostatic potential. This situation allowed the creation of a highly directional attractive interaction with a negative site. Clark supported its model in the results of Brinck [36] on surface electrostatic potentials of halogenated methanes, that already pointed to the existence of regions of positive electrostatic potential on the outer surfaces of halogen atoms in RX molecules, with the rest of the atom surface displaying negative potential. The participation of halogen bonds between halogen atoms in covalent molecules and negative sites in similar or different molecules has been widely reported in the literature [37,38,39]. More recently it was demonstrated theoretically, as well as observed experimentally, that similar interactions could exist between Groups 14, 15 and 16 atoms and nucleophiles, due to the presence of a σ-hole in those atoms [40,41,42]. These interactions follow the same trends in electronegativity and polarizability than those observed for halogens. 

Today halogen contacts are well known as responsible for the supramolecular arrangements of a large number of organic, organometallic and biological molecules [16,43,44,45,46,47,48].

**Scheme 3:**
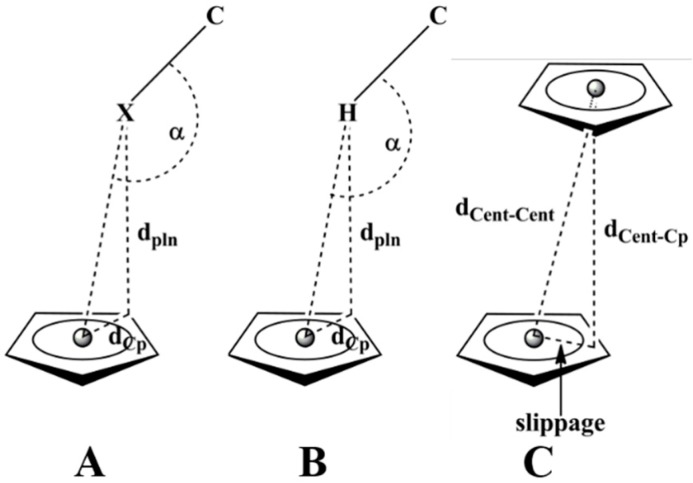
(**A**) C-X⋯π interactions; (**B**) C-H⋯π interactions; (**C**) π-π interactions (parameters adapted from Nishio [60]).

Another type of halogen bond that received attention in the literature recently are C-X⋯π interactions (X = Cl, Br) (Scheme 3A) [49,50]. They are present in systems containing both halogen atoms and aromatic rings, such as ligand-protein [51,52] and nanomaterial carbon systems [53,54]. Their main driving force is the electrostatic interaction between the σ-hole on the top of the halogen atom and the aromatic electron cloud. This electrostatic attraction increases with X polarizability and this type of contact can be classified as a halogen bond because the halogen atom is an electron acceptor. Directionality affects the contribution of dispersion forces to these bonds and they can become more dominant the further the molecules move away from an axial configuration, with the C-X bond pointing directly to the aromatic electron cloud. Theoretical calculations have proved that even when electrostatic interaction component is weaker than the dispersion energy it is still comparable in magnitude to the overall interaction energy [55,56,57,58,59].

The presence of π rings also creates the possibility for C-H⋯π and π-π stacking interactions (Scheme 3B,C). Reports on attractive interactions between a C-H bond and an aromatic ring were presented in the early 50’s [61] but it was not until the late 1970’s that Nishio and co-workers looked with further detail into this type of hydrogen bond [60].

Several studies on the geometrical characteristics [62,63] and the structural effects of these interactions have been published [64,65,66,67]. Together with other authors Nishio proposed that these C-H⋯π interactions were the result mainly of dispersion forces, particularly where the donor carbon atom presented sp^3^ or sp^2^ hybridization [64,68]. However, these interactions are also affected by electrostatic factors resulting from dipole-quadrupole and charge transfer interactions that affect their directionality [64]. Their energy depends both on the donating capacity of the C-H group and the electron density of the π system. 

C-H⋯π interactions very often contribute in defining the conformation and crystal structure of organic compounds (making use of intra- and intermolecular interactions, respectively), but they also play a relevant role in the supramolecular structures of organometallic [69], and biomacromolecular compounds [62,64].

π-π stacking interactions involve approximately parallel aromatic molecules with an interplanar distance between 3.3 and 3.8 Å [70]; their main energetic contributions are also dispersion and electrostatic forces, although the role played by the last ones is still being debated [71]. Apart from their relevance in defining supramolecular structures of organic and organometallic compounds, π-π interactions are fundamental in promoting drug interactions with DNA and proteins and enabling electric conduction between aromatic organic systems [70].

In the 1950’s the first evidences of D-H⋯M interactions were reported in ferrocenyl alcohols [72]. Only in the 70’s decade they attracted again the attention of hydrogen bond motivated scholars [73,74,75]. The majority of the examples found in the literature refer to intramolecular interactions [76,77,78,79,80] but several examples of intermolecular Y-H⋯M contacts have also been reported [14,77,81,82,83]. Braga et al. [83] determined that intermolecular Y-H⋯M hydrogen bonds involve sterically available electron-rich low oxidation state late transition metals and show H⋯M distances smaller than D⋯M distances and D-H⋯M angles larger than 100°.

Borissova et al. [14] studied the role played by D-H⋯M hydrogen bonds in orthorhombic ruthenocene, creating well-ordered chains and at the same time constraining the rotation of the Cp rings, justifying a lower ring disorder for these compounds than for other Group VIII metallocenes in monoclinic and triclinic space groups.

The aim of this work is to study the crystal packings of halometallocenes and how the various types of intermolecular interactions previously described affect them. A particular interest will involve the competition between X⋯X and X⋯H contacts, as well as the type of halogen contacts formed in these molecules. Comparison between the results encountered in this work with those from a similar one on the supramolecular arrangements and interactions in di-halogenated bent metallocenes [16] will show the effect of the halogen positioning in the electrostatic potential distribution around the molecule, and how these changes will influence the type of halogen interactions observed, conditioning the crystal packings formed in these two families of compounds.

## 2. Results

This study is based on a search in the Cambridge Structural Database (CSD version 5.38) [84] on Group VIII 1,1′-halometalocenes that resulted in 6 hits (Table 1). One of the entries for (CpCl)_2_Fe (DUTSUH) did not include data on coordinates, so it was excluded.

The main text includes the discussion of the crystal packing and the most relevant parameters for that discussion (for atom numbering see Scheme 1). Full data associated with the interactions reported in the paper can be found in the Electronic Supplementary Information (ESI) (see Tables S1 to S6).

### 2.1. Electrostatic Potentials by DFT Calculations

**Figure 1:**
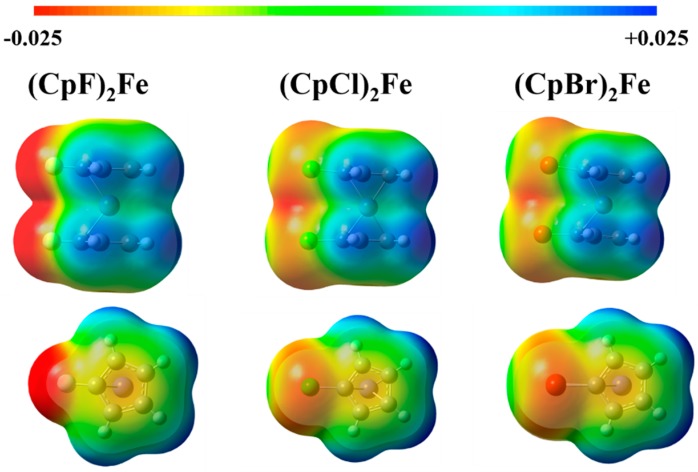
Electrostatic potential mapped onto an electron density isosurface for (CpX)_2_Fe (side and top views, X = F, Cl, Br). Positively or negatively charged regions are indicated by colour gradients changing from blue to red, respectively (scale in atomic charge unit/Å^2^). The isosurface depicted in this and the following map figure correspond to the isodensity of 0.0004 relative to the total electron density.

**Figure 2:**
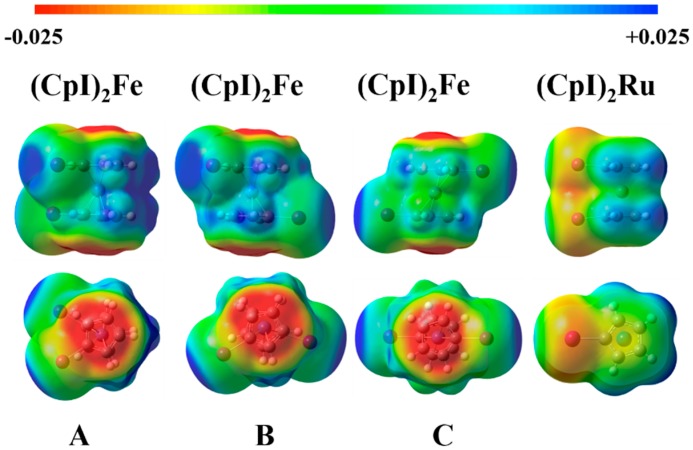
Electrostatic potential mapped onto an electron density isosurface for three isomers of (CpI)_2_Fe and for (CpI)_2_Ru (side and top views). The isomers present, respectibly, angles of 60, 123 and 180° between the C-I bonds.

DFT (Density Functional Theory) calculations were performed to obtain electrostatic potentials mapped onto electron density isosurfaces (Figure 1 and Figure 2; the same electrostatic potential scale is used for all the structures). They also allowed the construction of a table of point charge values (Table S1) for the various atoms in the compounds. Both were used to analyze the factors influencing the presence of the various types of noncovalent interactions.

### 2.2. Analysis of Interactions Recurring to Hirshfeld Surfaces

**Figure 3:**
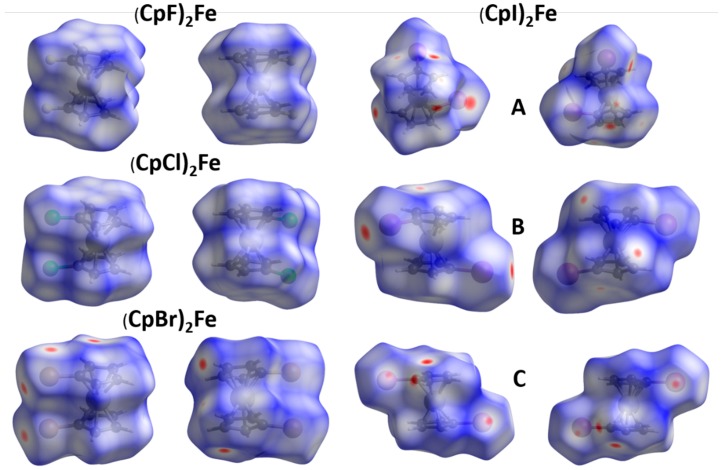
Hirshfeld surfaces for (CpX)_2_Fe compounds (X = F, Cl, Br, I (three isomers)).

**Figure 4:**
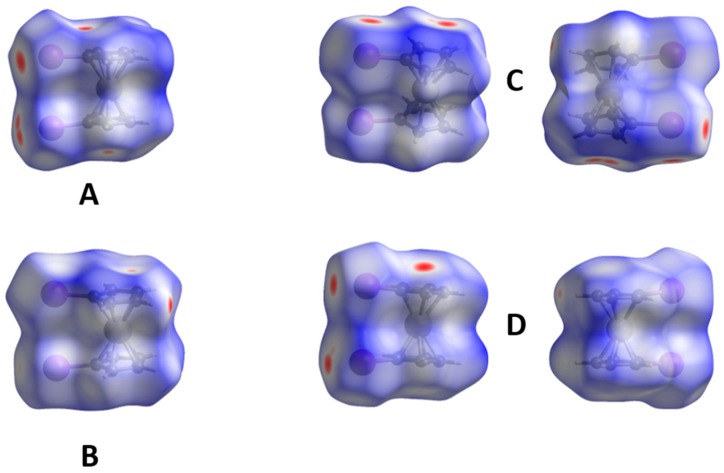
Hirshfeld surfaces for the four inequivalent molecules in the asymmetric unit of (CpI)_2_Ru.

The 3D *d_norm_* Hirshfeld surface plots of the five complexes included in this study are shown in Figure 3 and Figure 4. The large and deep red spots on the 3D Hirshfeld surfaces indicate the areas where the close-contact interactions take place. The percentage contributions of the various types of interaction are indicated in Table 2.

According to the numerical results of the Hirshfeld analysis for all the molecules the greatest percentage of intermolecular contacts is related to the proximity induced H⋯H contacts. The other types of contacts present will be analyzed case by case in the next section, together with crystal packing diagrams of the supramolecular arrangements that they produce. 

### 2.3. Supramolecular Arrangements

To select the interactions included in this study the authors used Mercury software (see Section 4.1. X-Ray crystallographic analysis) to detect supramolecular features responsible for defining the crystal packing of the compounds. The interactions obtained using Mercury default parameters need to be scrutinized in order to remove pseudo contacts with a distance shorter than the sum of the van der Waals radii; when important patterns detected in the crystal packings could not be explained by contacts shorter than the sum of the van der Waals radii the cut-off distance was extended 0.4 Å, based on the criteria proposed by Dance [89]. According to this author such interactions would fall in the potential energy zone where attractive interactions are predominant. This situation was mainly applied to D-H⋯M hydrogen bonds, taking into consideration the uncertainty in the values of van der Waals radii of metals, a result of the fluctuations in charge that they present in organometallic compounds [14]. 

Hirshfeld surface analysis was also used to support the choices made.

### 2.4. 1,1′-Difluoroferrocene and 1,1′-Dichloroferrocene

Both molecules show an eclipsed molecular structure [85]. The charge distributions show negative areas around the halogen atoms (more pronounced for fluorine and with a small σ-hole in the case of chlorine) and over the Cp rings, with positive charges covering the hydrogen atoms (in Figure 1 and Table S1 it is possible to detect that the positive charges are slightly higher in H2(7) and H5(10) for fluorine than in H3(8) and H4(9) for chlorine).

Despite not showing well pronounced areas resulting from contacts on the Hirshfeld surface plots (Figure 3), the statistical results (Table 2) predict that the most significant contacts in these two molecules would be C-H⋯X (X = F, Cl) hydrogen bonds and π-π interactions.

**Figure 5:**
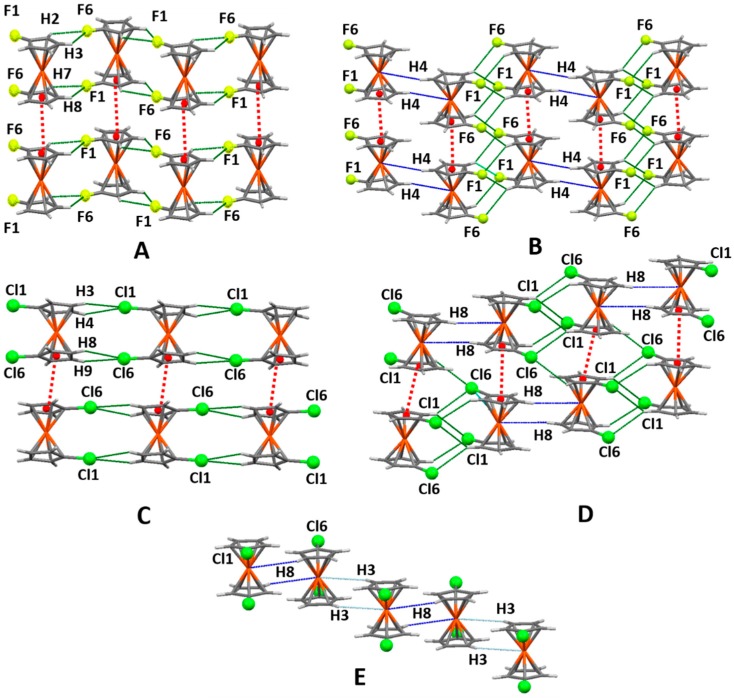
View of supramolecular arrangements of (CpF)_2_Fe (planes 0,1,6 (**A**), and 0,0,1 (**B**)) and (CpCl)_2_Fe (planes 1,0,1 (**C**), 1,0,1 (**D**) and 1,0,0 (**E**).

The primary supramolecular arrangements of these two compounds look very similar (Figure 5A,C). They both organize in head to tail molecular chains formed by double C-H⋯X chelated hydrogens bonds. The C-H⋯X bonds display small angle values (113–118.5°, see Tables S2 and S3), not very common in classical hydrogen bonds, but that have already been found in other examples involving C(sp^2^)-H⋯Cl contacts [90]. 

But these chains also present significant differences. In the chlorine compound the molecules have the same orientation along the chain, while in the fluorine compound the molecules reverse their ring position. In both compounds the chains interact among themselves by π-π interactions between rings I-I and II-II for fluorine and II-II for chlorine (centroid-centroid distances of 3.5–3.6 Å), but with fluorine the halogen atoms in all the chains point in the same direction, while with chlorine they point in one direction in the top chain and reverse their orientation in the chain below.

Even more striking is the change in hydrogen atoms involved in the C-H⋯X bonds: H2(7) and H3(8) for fluorine and H3(8) and H4(9) for chlorine. This results in different alignments for the molecules: zigzag chains and linear chains, respectively, a consequence of the high electronegativity of fluorine, that extends through the aromatic ring, making the adjacent atoms H2 and H7 one of the most positive combination of hydrogens in the Cp groups, while with chloride H3-H8 and H4-H9 show the highest values of positive charge (see Table S1). 

Another motif formed in the crystal packing of these two compounds are dimers composed by rotated molecules recurring to C-H⋯X bonds (Figure 5B,D). The dimers form vertical chains also using C-H⋯X contacts reinforced by I-II ring π-π interactions for F and I-I and II-II interactions in the case of Cl. 

There are three-dimensional evidences in the fluorine compound that the dimer chains are linked by double C4-H4⋯Fe bonds, forming zigzag horizontal chains. A similar feature was detected for the chlorine compounds, but this time using C8-H8⋯Fe bonds and resulting in a linear chain. The recognition of these interactions requires an increase in the Mercury distance tolerance of 0.3 Å, within the criteria proposed by Dance [89]. Despite not showing in the Hirshfeld analysis (which is based on a zero tolerance in the sum of van der Waals radii), its existence is supported by the presence of the chains and their high directionality. In the chlorine compound a second H⋯M bond (C3-H3⋯Fe, at an angle of 136.43° with C8-H8⋯Fe) is also formed, creating another chain of rotated molecules (Figure 5E). This chain forms a 2D arrangement via I-I and II-II π-π interactions.

### 2.5. 1,1′-Dibromoferrocene

This molecule also shows an eclipsed conformation and a very similar charge distribution to (CpCl)_2_Fe; the main difference is the slightly less negative charge of the halogen (Table S1) and the larger σ-hole that this atom presents (Figure 1). Hirshfeld analysis predicts the presence of Br⋯Br, C⋯Br (C-Br⋯π), and C⋯H (C-H⋯π) contacts, while the relevance of C-H⋯Br hydrogen bonds is maintained and π ⋯π interactions vanish. All these interactions involving bromine atoms and Cp rings show in the Hirshfeld surfaces with the presence of red spots in areas surrounding these groups (Figure 3).

**Figure 6:**
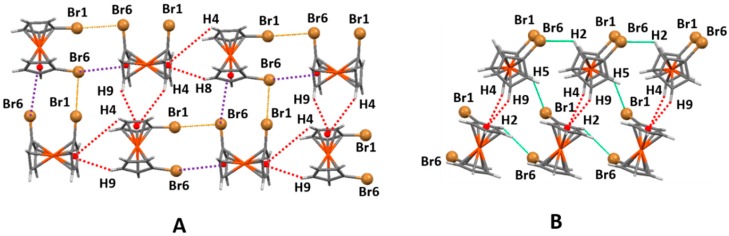
View of supramolecular arrangements of (CpBr)_2_Fe (planes 1,0,1 (**A**), and 0,−1,1 (**B**)).

A packing completely different from the previous two is expected. Initially it forms 2D motifs that are based in Br1⋯Br6 halogens bonds (Figure 6A). The geometry of these interactions forces the molecules to rotate 90°, so they get involved in two other types of interactions: C6-Br6⋯π and a chelated double C4-H4⋯π and C9-H9⋯π contact. 

The second motif (Figure 6B) is near perpendicular to the first and it involves molecular chains united by C2-H2⋯Br6 interactions. These chains form interchain C1-Br1⋯H5 bonds, reinforced by the same C-H⋯π contacts.

The presence of halogens bonds and C-Br⋯π contacts is a situation that results from the increase in size of the σ-hole, allied to the decrease in electronic charge of the halogen atom, making it more positive and therefore more available to accept electronic density from the negative region of another halogen atom or from an aromatic π group.

The hydrogen atoms of the ring are also slightly less positive due to the decrease in electronegativity of the halogen atom, reducing the proton donation capability of the C-H bond; meanwhile there is a small increase in the positive charge of the metal atom. Both conditions, together with the geometry of the dominant interactions, affect the capability to form C-H⋯M bonds [68].

### 2.6. 1,1′-Diiodoferrocene

Despite developing a method that allowed to obtain this compound in high yields and great purity (>99.9%), the authors of the only report on the crystal structure of 1,1′-diiodoferrocene [87] only managed to obtain a final product that was a mixture of three isomers: isomers A and B, with an eclipsed conformation and with C-I bonds forming angles of 60° (A) and 123° (B) between each other, and isomer C with a staggered conformation and C-I bonds at an angle of 180°. The absence of an eclipsed conformation with C-I bonds on top of each other must be the result of the large volume of the iodine atom, that contributes to restrict the rotation of the Cp rings, blocking them and forming the three isomers. The deviation of the halogen atoms from the eclipsed conformation affects the orbitals involved in the metal/ligand electron flow (see Figures S1 and S2), causing a charge distribution completely different from those observed in the previous compounds, namely a very large concentration of positive charge in the iron atom (224–265 a.c.u.%—atomic charge units percentage—see Table S1). These values show a large contribution of metal back bonding in those molecules, also contributing for the larger negative charges in the carbons of the Cp rings (Figure 2). 

The simultaneous presence of these three isomers makes the crystal packing very complex, so it was decided to display only the main interactions encountered, instead of trying to generate intricate geometrical patterns that would not help to systematize the effect of the presence and positioning of halogen atoms in the crystal packing. The Hirshfeld analysis predicts a considerable increase in relevance of I⋯I, and C-H⋯π interactions, while only slightly decreasing the presence of C-I⋯H contacts (Table 2); these results also show in the Hirshfeld surfaces, with a substantial increase of red spots near halogen atoms, rings and hydrogen atoms (Figure 3).

**Figure 7:**
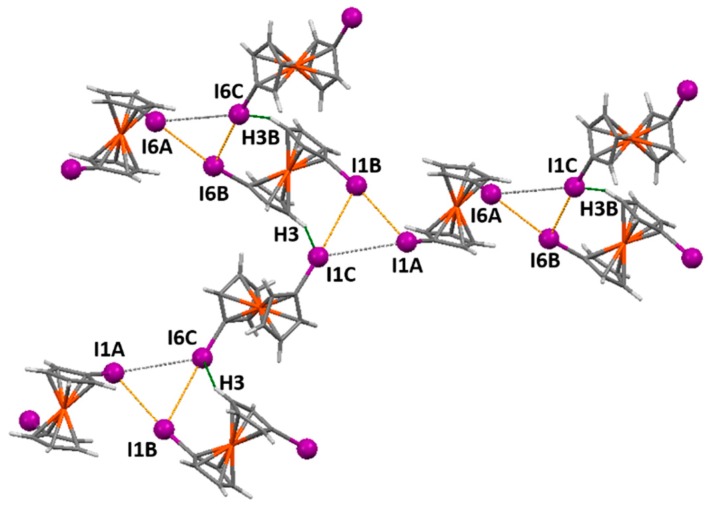
View of part of supramolecular arrangements of (CpI)_2_Fe (halogen bonds in orange, hypothetical type I halogen contacts in grey and C-H⋯I bonds in green).

The synthon which geometrically dominates this compound supramolecular pattern is a V-shaped double I⋯I halogen bond, a consequence of the increase in the σ-hole and the slightly positive charge of the halogen atom. It involves interactions between iodine atoms from isomers A and B and from isomers B and C (in orange in Figure 7). The θ_1_ and θ_2_ angles observed for C-Br⋯Br (see Scheme 2; 172.41°, 95.70° for A⋯B interactions; 169.55°, 109.66° for B⋯C interactions) classify these interactions as halogen bonds, with all parameters within the expected values [45]. However, if the Mercury program tolerance for contact distances in enlarged by 0.3 Å a third I⋯I interaction is detected, this time between iodine atoms of isomers A and C (in grey in Figure 7); its parameters (θ_1_ = 131.09°, θ_2_ = 131.17°) make it not a halogen bond but a Type I halogen contact. The combination of these three interactions is, apart from the differences in angle values, comparable to halogen trimers already reported in the literature [26,91]. Apart from this “trimer” the crystal pattern is reinforced by several individual and multifurcated C-H⋯I bonds (see Table S5). For reasons of clarity only three are pictured in Figure 7. For (CpI)_2_Fe a particular situation occurs, due to the charge inversion suffered by the hydrogen atoms, that present negative values. This negative character can facilitate the interaction between the hydrogens and the positive σ-hole of the iodine atoms, explaining the average increase in the angles observed for C-H⋯I bonds when compared to those displayed by interactions involving lighter halogens (see Tables S2, S3 and S5).

The large increase in charge observed in the ring carbons can account for the increase in C-H⋯π interactions in similar percentages to the bromine compound. This is a circumstance where dispersive forces are more relevant in this type of interactions than electrostatic forces, and they should increase accordingly to the growth of negative charge in the rings.

### 2.7. 1,1′-Diiodoruthenocene

This compound crystallizes with 4 inequivalent molecules in its asymmetric unit (A, B, C, D). The differences between them are minimal and they all present an eclipsed conformation with over positioning of the iodine atoms. 

**Figure 8:**
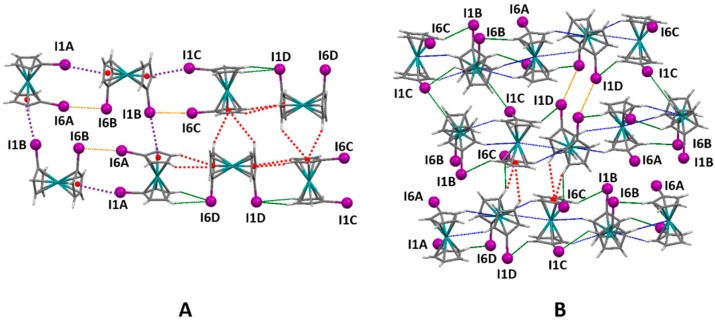
View of supramolecular arrangements of (CpI)_2_Ru (planes −1,0,3 (**A**) and 1,−1,1 (**B**).

An analysis of Figure 1 and Figure 2 and Table S1 shows that charge distribution in (CpI)_2_Ru is very similar to the one observed in (CpBr)_2_Fe. Therefore, it is not surprising that the two crystalline primary patterns are very similar (Figure 6A and Figure 8A). However, there are some important differences in the Hirshfeld analysis of the interactions in these two compounds (Table 2).

The increase in percentage for the I⋯I and C-I⋯π interactions is not surprising, as it results again from the increase in σ-hole size and decrease in electronic charge of the halogen atom. The growth of the last type of interactions is not as significative as in (CpI)_2_Fe because the negative charge of the rings does not increase so considerably. I⋯I and C-H⋯I interactions are observed this time both in the primary and the secondary patterns (Figure 7B). The main difference encountered when compared with (CpBr)_2_Fe and (CpI)_2_Fe is the presence of C-H⋯Ru bonds, a result of the larger inter-ring distance observed for Ru (average 3.626 Å for (CpI)_2_Ru and 3.286 Å for (CpI)_2_Fe), that facilitates the contact between C-H bonds and the metal atom and, together with the more diffuse character of the metal d orbitals, compensates for the low proton donation capability of those bonds.

## 3. Discussion

The crystal packing both in group VIII 1,1′-halometallocenes (M(C_5_H_4_X)_2_ and group IV di-halogenated bent metallocenes (Cp_2_MX_2_) [16] is affected in different ways by the competition between X⋯X halogen interactions and C-H⋯X hydrogen bonding. 

**Figure 9:**
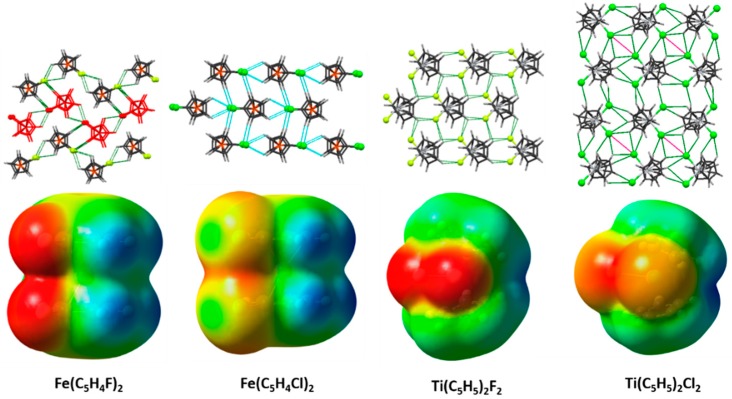
Primary supramolecular arrangements of Fe(C_5_H_4_X)_2_, and Ti(C_5_H_5_)X_2_ (X = F, Cl) (**top**); electrostatic potential mapped onto an electron density isosurface for the same compounds (**bottom**).

The weak hydrogen bonds are the dominant feature when more electronegative halogen atoms (fluorine and chlorine) are involved (Figure 9). The presence of a large negative concentration in the front part (the halogen atoms) and a positive site in the opposite side of the molecule (the C-H bonds) facilitates the formation of head to tail chains of molecules through C-H⋯X hydrogen bonds.

Despite these similarities there are relevant differences between the supramolecular organization of these two classes of halometallocenes. In Fe(C_5_H_4_F)_2_ the chain adopts a zigzag antiparallel alignment, caused by the high electronegativity of fluorine, that, as a Cp substituent, induces very positive atomic point charges in the adjacent hydrogen atoms. The bonding of the fluorine directly to the metal and its higher negative atomic point charges in Ti(C_5_H_5_)F_2_ (−45 a.c.u.% [14], −20 a.c.u.% in Fe(C_5_H_4_F)_2_, see Table S1), favors not only a linear geometry but also a parallel alignment of the chains.

As expected, both fluorine compounds do not exhibit halogen interactions. The chlorine compounds, with a halogen atom of less electronegativity and higher polarizability, could both present such contacts. However they are detected in Ti(C_5_H_5_)Cl_2_ but not in Fe(C_5_H_4_Cl)_2_, even with both displaying similar linear and antiparallel chains. An analysis of the electrostatic potential maps in the lower part of Figure 9 shows that while in the Fe compound the Cl atoms are located one above the other, pointing in the direction of the progression of the chains, in the Ti compound the chlorine atoms are bonded directly to the metal, forming an angle between Ti-Cl bonds. This situation, and the antiparallel nature of the chains, leaves them facing chlorine atoms in other molecules, enabling the formation of type I halogen interactions. The fact that only some of the possible Cl⋯Cl contacts are formed results from the existence of two inequivalent molecules in the asymmetric unit that cause significant differences in the intermolecular distances between various types of Cl atoms [16].

Further decrease in electronegativity along the halogen group, accompanied by an increase in polarizability, favours a growth in the positive σ-hole on top of bromine and iodine. This should result in a rise in relevance of halogen-halogen contacts compared to hydrogen bonds. However, Hirsfeld analysis shows that hydrogen bonds are present for all compounds in a similar percentage (see Table 2 and reference [16]), probably because of statistical reasonings concerning the larger number of H atoms present in the molecules (eight) when compared to the number of halogen atoms.

**Figure 10:**
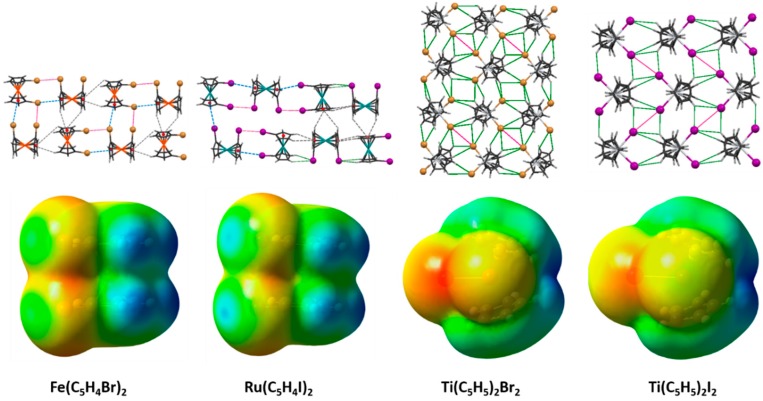
Primary supramolecular arrangements of Fe(C_5_H_4_Br)_2_, Ru(C_5_H_4_I)_2_ and Ti(C_5_H_5_)X_2_ (X = Br, I) (**top**); electrostatic potential mapped onto an electron density isosurface for the same compounds (**bottom**).

The packing in bromine and iodine 1,1′-halometalocenes shows that hydrogen bonds no longer define the main features of the crystal packings (Figure 10). The main reason for the important role played by halogen interactions is the presence of Type II halogen bonds that were inexistent in biscyclopentadienyl dihalides where the halogen atoms form two V-shaped metal–halogen bonds concentrating the negative electrostatic potential in the zone amidst those bonds, disabling this option (see electrostatic potential maps in Figure 10). In Fe(C_5_H_4_Br)_2_, Fe(C_5_H_4_I)_2_ and Ru(C_5_H_4_I)_2_ this negative region is more exposed and facilitates the formation of halogen bonds involving this area with electron donor capabilities and the σ-hole acting as an acceptor.

In di-halogenated bent metallocenes, despite the increase in percentage in the Hirshfeld analysis of halogen contacts, the supramolecular networks (Figure 10) are still formed by head to tail chains of molecules through C–H⋯X hydrogen bonds. As discussed above, the location of the Ti-X bonds reinforces this type of array due the formation of Type I halogen interactions. For the same reason presented for Ti(C_5_H_5_)Cl_2_ only some of this contacts are present in Ti(C_5_H_5_)Br_2_, while in Ti(C_5_H_5_)I _2_ (with a single highly symmetrical molecule in the asymmetric unit) all I atoms are involved.

Another type of halogen bonds that participates in the definition of the crystalline frameworks in 1,1′-halometalocenes are C-X⋯π interactions. They are observed in Fe(CpBr)_2_ and Ru(CpI)_2_, reporting angles between the C-X bond and the projection of the halogen atom on the ring plane that are considerably large (152.8–173.14°). This fact, together with the larger size of the halogen σ-hole, indicate that the electrostatic interaction with the aromatic electron cloud can be the predominant factor in their genesis. The absence of this type of interactions in (CpI)_2_Fe is due to the presence of three isomers in the crystalline mixture and their predominant halogen-halogen interaction patterns; these molecules show a larger electron density in the aromatic ring that could enhance the effect of a dispersion contribution.

Pattern evidences of the presence of C-H⋯M bonds were encountered in Fe(CpF)_2_, Fe(CpCl)_2_, and Ru (CpI)_2_. In the first two compounds these interactions are enhanced by the large proton donation capability of the C-H bonds, while in the ruthenium compound the longer distance between the Cp rings helps the penetration of the molecules. Because of the location of the halogen atoms they cannot be observed in di-halogenated bent metallocenes.

This work allows to compare two families of compounds with similar composition but different molecular bonding arrangements, leading to large differences in the electron density distribution. In the supramolecular networks of these molecules the dominant contacts are weak hydrogen bonds and halogen interactions. Although it is widely accepted that halogen bonding probability increases along with the polarizability of the halogen atom, these results indicate that this does not always correspond to a greater relevance in the definition of the crystal packings of the molecules, even if they are competing with weak hydrogen bonds. Apart from electronegativity and polarizability, because of its high directionality, halogen contacts are also very much affected by electron density distribution on the molecules. This effect is felt on their spatial orientation and, consequently, on the type of halogen interactions formed (namely Type I halogen contacts or Type II halogen bonds). With the proper orientation the halogen contacts formed may even tend to reinforce the supramolecular arrangements ruled by hydrogen bond networking instead of taking control of the global crystalline structure.

## 4. Methods 

### 4.1. X-ray Crystallographic Analysis

Intramolecular and intermolecular interactions were analysed using Mercury CSD 3.0 (Build RC5) [92]. This programme allows the visualization of 3D crystal packings based on files containing atom coordinates (like *.cif, *.res, etc.) as well as to locate intermolecular and/or intramolecular hydrogen bonds and short nonbonded contacts in a given distance range, in terms of van der Waals corrected distances. It also allows building and visualizing a network of intermolecular contacts. The parameters for these contacts can be defined by the user, regardless of the software using default definition of a short contact as any intermolecular contact shorter than the sum of the van der Waals Radii and of a hydrogen bond as an interaction involving an hydrogen atom with a donor⋯acceptor distance smaller than the sum of van der Waals radii of these two atoms.

The interactions proposed by Mercury need to be scrutinized in order to remove pseudo contacts with a distance shorter than the sum of the van der Waals radii (or of a similar magnitude) that are actually proximity induced contacts like H⋯H. It is also necessary to be aware of important patterns presented in the crystal packing that apparently have no detectable interactions in their genesis. In such a situation it is possible to extend the cut-off distance to find such interactions, as it was done in this work up to 0.4 Å, based on the criteria proposed by Dance [89]. According to this author such interactions would fall in the potential energy zone where attractive interactions are predominant.

### 4.2. Ab Initio Calculations

Electrostatic charge distributions were calculated using Gaussian 03 [93] at the B3LYP [94] level of theory. The SDD basis set [95] with f-polarization functions was used for the transition metals and iodide atoms and the 6-311G(3df,3pd) basis set [96,97] for the carbon, hydrogen and halogens atoms. The point charges placed at the center of mass of each atom of the molecules are then calculated from the electronic density function using an electrostatic surface potential methodology (CHelpG) [98]. Single point calculations were performed using the geometries obtained from the crystallographic data of the isolated molecules.

### 4.3. Hirshfeld Surface Calculations

Molecular Hirshfeld surface calculations were performed using the Crystal Explorer program (version 3.1) [99]. In this study, all the Hirshfeld surfaces were generated using a high (standard) surface resolution. The 3-D *d_norm_* surfaces [100] were mapped using a fixed color scale of −0.0964 (red) to 0.9305 (blue).

## Figures and Tables

**Table 1:** Results from the CSD search on group VIII 1,1′-halometalocenes.

Compound	Ref.-Year of Publication	CSD Refcode		Space Group	Z	Z′	Temp. (K)	R-Factor (%)
(CpF)_2_Fe	[85]-2015	RACROF	Monoclinic	P21/n	4	1	173	2.96
(CpCl)_2_Fe	[84]-1986	DUTSUH	Monoclinic	C2/c	4	0 ^a^	295	4.60 ^a^
(CpCl)_2_Fe	[85]-2015	DUTSUH01	Monoclinic	C2/c	4	1	173	2.00
(CpBr)_2_Fe	[86]-2004	BIPDOU	Monoclinic	P21	2	1	100	3.67
(CpI)_2_Fe	[87]-2014	KOPFAY	Monoclinic	C2/c	12	3	100	2.20
(CpI)_2_Ru	[88]-2014	JODWOQ	Triclinic	P-1	8	4	183	4.1

^a.^ No coordinates included.

**Table 2:** Contribution (in percentage) to the Hirshfeld surface area of the various intermolecular contacts.

Contact	(CpF)_2_Fe	(CpCl)_2_Fe	(CpBr)_2_Fe	(CpI)_2_Fe (A)	(CpI)_2_Fe (B)	(CpI)_2_Fe (C)	(CpI)_2_Ru (Average)
X-X	1	0.6	2.3	11.3	13.7	8.9	7.2
X-C	0.2	1.9	3.7	0.8	1.1	0	8.7
X-H	36.8	40.6	39.6	36	33.3	29.3	33.7
X-M	0	0	0	0	0	0	0
C-C	9.1	8.5	0	2.9	2.9	0	0.9
C-H	3.6	3.2	17.1	25.5	8.6	13.7	13.4
C-M	0	0	0	0	0	0	0
H-H	49.3	45.2	37.3	23.5	40.4	48.1	35.8
H-M	0	0	0	0	0	0	0.2

## Supplementary Materials

The following are available online at http://www.mdpi.com/1420-3049/23/11/2959/s1, Figure S1. Side and top view of HOMO molecular orbitals surfaces for (CpX)_2_Fe (X = F, Cl, Br), Figure S2. Side and top view of HOMO molecular orbitals surfaces for (CpI)_2_M (M = Fe (isomers A, B, C), Ru), Table S1. Atomic point charges (in atomic charge units percentage, a.c.u.%) for molecules (CpX)_2_M (M = Fe; X = F, Cl, Br, I. M = Ru; X = H), Table S2. Intermolecular contact parameters in (CpF)_2_Fe, Table S3. Intermolecular contact parameters in (CpCl)_2_Fe, Table S4. Intermolecular contact parameters in (CpBr)_2_Fe, Table S5. Intermolecular contact parameters in (CpI)_2_Fe, Table S6. Intermolecular contact parameters in (CpI)_2_Ru.
