# Supplementary material for: Halogen and Hydrogen Bonding Interplay in the Crystal Packing of Halometallocenes
Source: Molecules. 2018 Nov 13;23(11):2959. doi: 10.3390/molecules23112959 (PMC6278450; doi:10.3390/molecules23112959)
Supplement: Supplementary file 1 [file molecules-23-02959-s001.pdf]

Electronic Supplementary Information

"Halogen and Hydrogen Bonding Interplay in the Crystal Packing of Halometallocenes"

Karina Shimizu and J.L. Ferreira da Silva

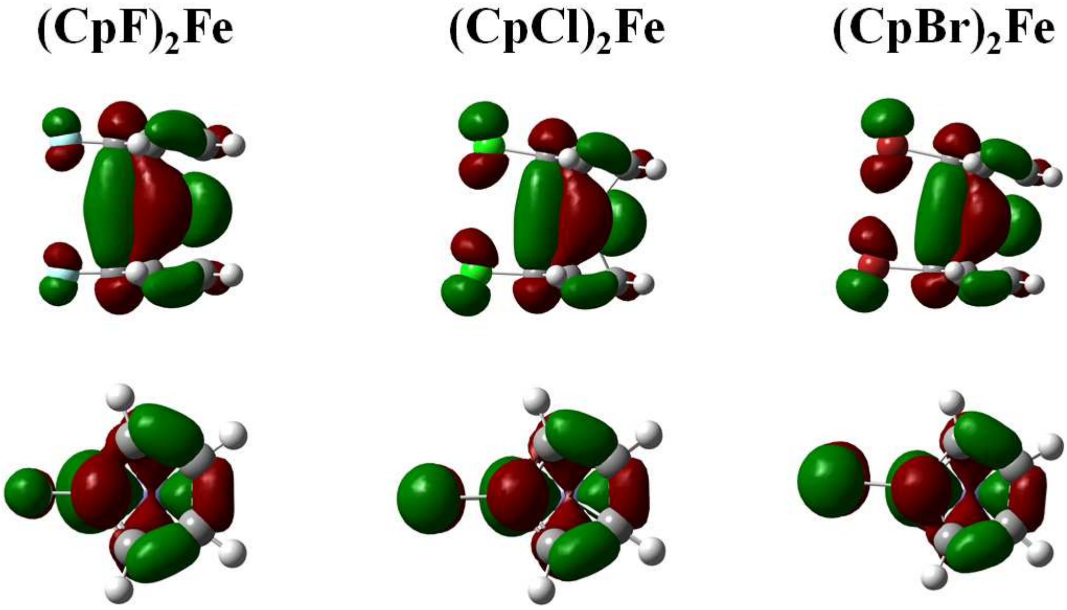

Figure S1 – Side and top view of HOMO molecular orbitals surfaces for $(CpX)_2Fe$ (X= F, Cl, Br).

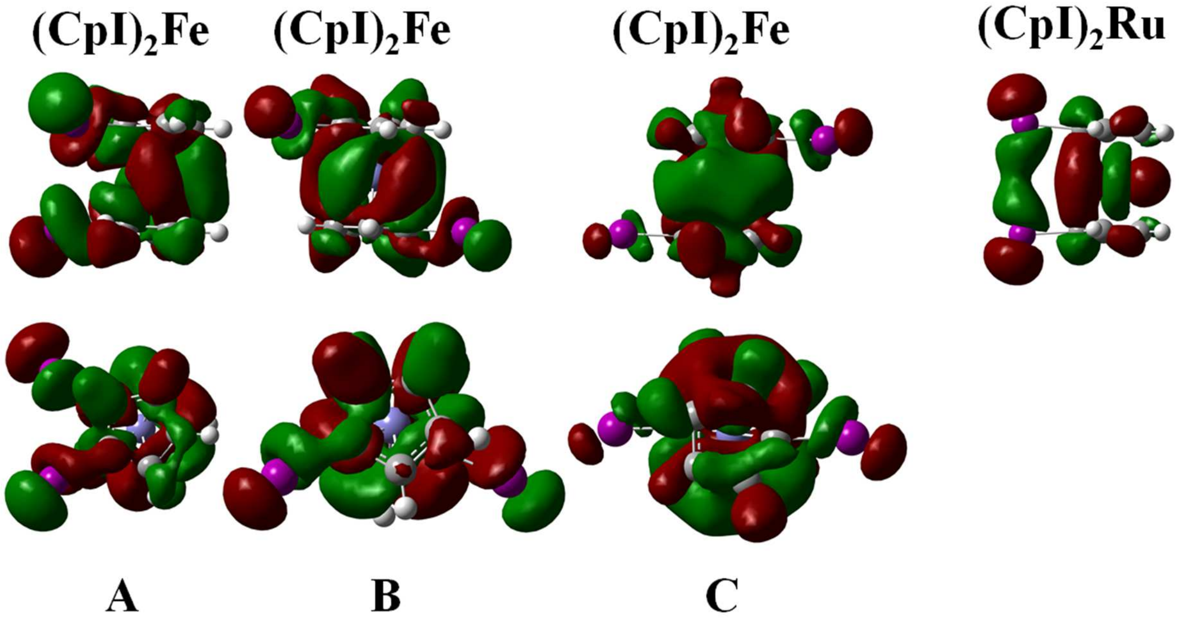

Figure S2 – Side and top view of HOMO molecular orbitals surfaces for $(CpI)_2M$ (M= Fe (isomers A, B, C), Ru).

Table S1 - Atomic point charges (in atomic charge units percentage, a.c.u.%) for molecules $(CpX)_2M$ (M = Fe; X = F,Cl,Br,I. M = Ru; X = H).

Compound	$(CpF)_2Fe$	$(CpCl)_2Fe$	$(CpBr)_2Fe$	$(CpI)_2Fe$[a]	$(CpI)_2Fe$[b]	$(CpI)_2Fe$[c]	$(CpI)_2Ru$
M	8	10	13	224	231	265	6
X1	-20	-12	-9	6	8	5	-6
C1	27	6	-1	-43	-47	-7	-3
C2	-17	-7	-6	6	0	-24	-1
C3	-15	-13	-13	-40	-16	-6	-11
C4	-13	-12	-13	-6	-29	-8	-11
C5	-19	-8	-6	-11	-2	-30	-2
H2	13	10	9	-14	-14	-5	6
H3	13	11	11	2	-7	-4	9
H4	12	11	11	-5	-1	-4	10
H5	14	10	9	-7	-8	-1	6
X6	-20	-12	-8	6	8	7	-6
C6	27	5	0	-43	-48	-8	-1
C7	-18	-8	-7	-11	-4	-28	-2
C8	-13	-12	-12	-6	-26	-14	-9
C9	-14	-12	-12	-40	-17	0	-13
C10	-17	-7	-7	6	2	-27	-3
H7	14	10	9	-7	-6	-1	6
H8	12	11	11	-5	-2	-2	9
H9	12	11	11	2	-7	-5	11
H10	13	10	10	-14	-15	-5	6

a – isomer with an angle of 60° between the C-I bonds.
b - isomer with an angle of 123° between the C-I bonds.
c – isomer with an angle of 180° between the C-I bonds.

Table S2 – Intermolecular contact parameters in $(CpF)_2Fe$.

**C-F···H bonds (chains)**			
	C···F (Å)	H···F (Å)	C-H···F (°)
C7-H7···F1	3.262	2.758	113.99
C8-H8···F1	3.240	2.691	117.32
C2-H2···F6	3.270	2.753	115.01
C3-H3···F6	3.265	2.725	116.73
**C-F···H bonds (dimers)** [a]			
	C···F (Å)	H···F (Å)	C-H···F (°)
C2(5)-H2(5)···F1	3.595	2.785	143.68
C7(10)-H7(10)···F1	3.590	2.779	143.80
C2(5)-H2(5)···F6	3.607	2.810	142.01
C7(10)-H7(10)···F6	3.534	2.771	137.81
**C-H···M interactions**			
C-H···M	C···M (Å)	H···M (Å)	C-H···M (°)
C4-H4···Fe	4.261	3.445	145.28
**π···π interactions**[a]			
	Cent-Cent (Å)	Cent-Cp (Å)	Slippage (Å)
πI(II)(I)···πI(II)	3.561	3.550	0.280
πI···πII	3.561	3.550	0.280

[a] – numbers between parenthesis refer to interactions generated by symmetry

Table S3 – Intermolecular contact parameters in $(CpCl)_2Fe$.

**C-Cl···H bonds (chains)**			
	C···Cl (Å)	H···Cl (Å)	C-H···Cl (°)
C3-H3···Cl1	3.561	2.973	118.52
C4-H4···Cl1	3.566	2.987	117.90
C8-H8···Cl6	3.566	2.987	117.90
C9-H9···Cl6	3.561	2.973	118.52
**C-Cl···H bonds (dimers)**			
	C···Cl (Å)	H···Cl (Å)	C-H···Cl (°)
C2(5)-H2(5)···Cl1	4.039	3.184	144.29
C7(10)-H7(10)···Cl1	3.881	2.982	150.13
C2-H2···Cl6	3.776	3.171	120.28
C5-H5···Cl6	3.969	3.094	146.86
**C-H···M interactions**			
C-H···M	C···M (Å)	H···M (Å)	C-H···M (°)
C8-H8···Fe	4,433	3.555	147.80
C3-H3···Fe	4,433	3.555	147.80
**π···π interactions[a]**			
	Cent-Cent (Å)	Cent-Cp (Å)	Slippage (Å)
πI(II)···πI(II)	3.597	3.521	0.736

[a] – numbers between parenthesis refer to interactions generated by symmetry

Table S4 – Intermolecular contact parameters in $(CpBr)_2Fe$.

**Halogen bonds**			
	X···X' (Å)	C-X···X' (°)	X···X'-C' (°)
Br1···Br6	3.586	153.12	89.73
**C-X···H bonds**			
	C···Br (Å)	H···Br (Å)	C-H···Br (°)
C6-Br6···H2	3.789	3.229	119.55
C1-Br1···H5	3.724	3.069	127.52
**C-X···π interactions[a]**			
	d_{pln} (Å)	d_{cp} (Å)	α (°)
C6-Br6···π	3.262	2.791	152.8
**C-H···π interactions[a]**			
	d_{pln} (Å)	d_{cp} (Å)	α (°)
C4-H4···π	2.385	2.577	168.5
C9-H9···π	2.758	1.152	158.4

[a]The criteria developed by Nishio and co-workers (see ref.52) to describe this type of interactions will be adopted in the current manuscript: D_{pln} – distance from the H(X) atom to the plane of the π ring system
D_{cp} – distance between the projection of the hydrogen(halogen) atom on the ring plane and the π system centroid
α – angle between the C-H(C-X) bond and the projection of the hydrogen atom on the ring plane

Table S5 – Intermolecular contact parameters in $(CpI)_2Fe$.

**Halogen bonds**[a]			
	X···X' (Å)	C-X···X' (°)	X···X'-C' (°)
I1(6)A···I1(6)B	3.774	172.41	95.70
I1B···I1(6)C	4.098	169.55	109.66
I6B···I1(6)C	4.098	169.55	109.66
**Halogen contacts**[a]			
	X···X' (Å)	C-X···X' (°)	X···X'-C' (°)
I1A···I1(6)C	4.242	131.09	131.17
I6A···I1(6)C	4.242	131.09	131.17
**C-X···H bonds**[a]			
	C···I (Å)	H···I (Å)	C-H···I (°)
C3C-H3C···I1(6)A	4.032	3.251	136.08
C7B-H7B···I1A	4.090	3.190	150.52
C5B-H5B···I6A	4.090	3.190	150.52
C3B-H3B···I1(6)C	4.075	3.079	173.57
C4B-H4B···I1(6)C	4.121	3.474	124.34
C10A-H10A···I1(6)C	3.982	3.089	149.24
**C-H···π interactions**[a]			
	d_{pln} (Å)	d_{cp} (Å)	α (°)
C8(9)A-H8(9)A···πC	2.560	1.933	159.38
C5(10)A-H5(10)A···πB	2.915	0.419	112.58
C10C-H10C···πA	2.505	1.891	138.85

[a] – atom numbers between parenthesis refer to interactions generated by symmetry

Table S6 – Intermolecular contact parameters in $(CpI)_2Ru$.

**Halogen bonds**			
	X···X' (Å)	C-X···X' (°)	X···X'-C' (°)
I6A···I6B	3.727	169.29	91.10
I1B···I6C	3.685	167.02	96.49
**Halogen contacts**[a]			
	X···X' (Å)	C-X···X' (°)	X···X'-C' (°)
I1D···I6D	3.949	138.43	134.23
**C-H···X bonds (primary chains)**			
	C···I (Å)	H···I (Å)	C-H···I (°)
C3C-H3C···I1D	3.823	3.193	125.32
C4C-H4C···I1D	3.913	3.375	118.03
C3A-H3A···I6D	3.924	3.394	117.44
C4A-H4A···I6D	3.827	3.194	125.57
**C-H···X bonds (secondary chains)** [a]			
C2(5)B-H2(5)B···I1A	4.015	3.300	133.55
C7(10)C-H7(10)C···I1B	4.078	3.347	135.25
C7(10)A-H7(10)A···I6B	4.017	3.305	133.29
C7(10)B-H7(10)B···I1C	3.996	3.309	139.91
C2(5)C-H2(5)C···I1D	4.126	3.287	148.36
C2(5)A-H2(5)A···I6D	4.188	3.336	150.32
C-H···X bonds (interchain)			
C(8)9D-H(8)9D···I6C	3.926	3.185	136.17
C3(4)B- H3(4)B···I1C	4.272	3.350	164.56
**C-X···π interactions**			
	d_{pln} (Å)	d_{cp} (Å)	α (°)
C1A-I1A···πII	3.341	3.094	173.14
C1B-I1B···πI	3.124	2.620	165.44
**C-H···π interactions**			
	d_{pln} (Å)	d_{cp} (Å)	α (°)
C8A-H8A···πD	2.814	0.990	132.52
C9A-H9A···πD	3.082	1.853	137.82
C8D-H8D···πC	2.704	1.439	2.136
C4D-H4D···πC	2.864	150.52	152.54
**C-H···M interactions**[a]			
	C···M (Å)	H···M (Å)	C-H···M (°)
C2(5)A-H2(5)A···RuB	4.267	3.386	155.25
C7(10)A-H7(10)A···RuD	4.197	3.315	155.46
C7(10)B-H7(10)B···RuA	4.172	3.281	157.04
C(2)5B-H(2)5B···RuC	4.160	3.281	154.81
C2(5)C-H2(5)C···RuB	4.146	3.258	156.26
C7(10)C-H7(10)C···RuD	4.170	3.312	151.22
C7(10)D-H7(10)D···RuC	4.138	3.269	152.90
C2(5)D-H2(5)D···RuA	4.236	3.361	153.93

[a] – atom numbers between parenthesis refer to interactions generated by symmetry
